# Developing a novel transitional care model for older emergency department patients and exploring the target population in Taiwan

**DOI:** 10.3389/fmed.2024.1444724

**Published:** 2024-12-16

**Authors:** Hung-Lin Hsu, Chung-Han Ho, Ying-Jia Lin, Kang-Ting Tsai, Pei-Chi Yang, Shu-Lien Hsu, An-Chi Peng, Chien-Chin Hsu, Sam Yu-Chieh Ho, Chien-Cheng Huang

**Affiliations:** ^1^Department of Emergency Medicine, Chi Mei Medical Center, Tainan, Taiwan; ^2^Department of Medical Research, Chi Mei Medical Center, Tainan, Taiwan; ^3^Department of Information Management, Southern Taiwan University of Science and Technology, Tainan, Taiwan; ^4^Division of Geriatrics and Gerontology, Department of Internal Medicine, Chi Mei Medical Center, Tainan, Taiwan; ^5^Department of Senior Services, Southern Taiwan University of Science and Technology, Tainan, Taiwan; ^6^Department of Nursing, Chi Mei Medical Center, Tainan, Taiwan; ^7^School of Nursing, China Medical University, Taichung City, Taiwan; ^8^School of Medicine, College of Medicine, National Sun Yat-sen University, Kaohsiung, Taiwan; ^9^Department of Emergency Medicine, Kaohsiung Medical University, Kaohsiung, Taiwan; ^10^Department of Environmental and Occupational Health, College of Medicine, National Cheng Kung University, Tainan, Taiwan

**Keywords:** emergency department, nurse, older population, social, transitional care

## Abstract

**Aim:**

Transitional care in the emergency department (ED) has the potential to improve outcomes for older patients, but the specific population benefits from it and impact in Taiwan remain unclear. Therefore, we conducted this study.

**Methods:**

An interdisciplinary team comprising emergency physicians, dedicated transitional care nurse (TCN), nurse practitioners, nurses, geriatricians, and social workers was established at a tertiary medical center. TCN conducted screenings of ED patients (≥75 years) awaiting hospitalization to identify those in need of social or care support and aid as required. The team held regular meetings to address transitional care challenges. A retrospective analysis was conducted, comparing patients who received transitional care with those who did not, between February 1, 2022, and October 31, 2022, followed by a three-month outcome assessment.

**Results:**

We successfully implemented a novel ED transitional care model, involving 183 patients with TCN assistance and 374 patients without. Compared to patients without TCN, those with TCN were older, had more underlying comorbidities, required more nasogastric feeding and Foley indwelling, and had higher rates of hospice and palliative care. The common needs for TCN included providing home care instructions to a foreign caregiver (38.4%), long-term care referral (30.0%), care instructions for family members of older adults in long-term care facilities (26.3%), social worker referral (3.2%), and home healthcare referral (2.1%). Follow-up analysis showed no significant outcome differences between the two cohorts.

**Conclusion:**

The model we implemented identified the population benefiting from this service. Despite the frailty of patients receiving TCN, their outcomes were not inferior, suggesting the potential benefits of TCN for this population. Further research is warranted.

## 1 Introduction

The global aging issue is a major concern, with the proportion of the world’s population aged 60 years and above projected to nearly double from 12 to 22% between 2015 and 2050 (1). Taiwan is experiencing rapid population aging, with the percentage of older individuals already reaching 18.3% in 2023 (2). Projections indicate that this figure is expected to surpass 20% by 2025 (2). Older individuals typically experience a higher prevalence of chronic and complex diseases compared to younger populations (3). Ensuring continuous care for older adults can enhance care quality while reducing the burden on their family members, caregivers, and society (3).

The emergency department (ED) serves as a crossroad connecting various healthcare settings such as home, long term care facilities, outpatient care, and hospitalization (4). It also serves as the primary entry point for older patients with urgent medical needs, making the management of the ED crucial to the outcomes of this population (4). The American College of Emergency Physicians recommends the implementation of effective transitional care (TC) to facilitate outpatient care following an ED evaluation (4).

In Taiwan, TC is typically provided through discharge planning after hospitalization, with little emphasis placed on initiating such care within the ED. Numerous models for ED TC have been proposed, including the GEDI WISE (Geriatric Emergency Department Innovations in Care through Workforce, Informatics, and Structural Enhancements) program in the United States (5, 6). The GEDI WISE program was designed to meet the complex needs of older adults in emergency settings (5, 6). At its core, GEDI WISE incorporates ED-based Transitional Care Nurse (TCN) interventions, which specifically target the needs of geriatric patients by assessing and addressing their cognitive, functional, and social support requirements (5, 6). TCNs act as dedicated care coordinators, working to avoid unnecessary hospital admissions and facilitating safe, effective discharges (5, 6). Evidence from studies has shown that TCN involvement reduces 30-day readmission rates, lowers immediate hospitalization risk, and brings considerable healthcare cost savings, highlighting their critical role in enhancing care quality and efficiency for aging populations (5, 6). However, it is important to consider the cultural and insurance differences between Western nations and Taiwan, as the applicability of Western models may be limited. In Taiwan, the family-centric culture places strong responsibility for elder care on families, with significant reliance on live-in foreign caregivers, unlike Western models that often depend on professional providers. Additionally, Taiwan’s National Health Insurance offers near-universal coverage, reducing financial barriers but necessitating more education and support for navigating long-term care services. Transitional care models must address these cultural and systemic differences to be effective. Therefore, our study aimed to implement a model for ED TC specifically tailored to the Taiwanese context, adapted from the existing evidence-based United States model (i.e., GEDI WISE). We sought to investigate the targeted population and evaluate the outcomes of this new model. Our primary objective was to bridge the existing knowledge gap and make a meaningful contribution to the development of effective and culturally appropriate TC strategies within Taiwan’s ED setting. Additionally, our findings may serve as a valuable reference for other nations seeking to enhance their TC practices.

## 2 Materials and methods

### 2.1 Study hospital

This study was conducted at the Chi Mei Medical Center (CMMC), a Southern Taiwan tertiary medical center with over 60 full-time attending physicians and residents who served >121,000 ED patients in 2019 (7). In 2016, the CMMC ED established a Chi Mei Integrated Geriatric Emergency Team, and subsequently launched Taiwan’s first Geriatric ED in 2019, with the objective of leading and enhancing geriatric care for the rapidly growing older population (8). The Geriatric ED at CMMC employed various studies and approaches to generate local data and formulate solutions targeted toward the older population in Taiwan, including emergency medical services in the older population (9), geriatric syndromes and hospice care needs (10), a novel comprehensive screening tool (Emergency Geriatric Assessment) (11), computer-based and pharmacist-assisted medication review (12), hospice and palliative care (13), computer-assisted home care referral (14), and computerized tool and interdisciplinary care for older patients with delirium (15).

### 2.2 Establishment of interdisciplinary team and ED TC model

First, an interdisciplinary team consisting of emergency physicians, a dedicated TCN, nurse practitioners, nurses, geriatricians, and social workers was established in Feb 2022 to initiate TC in the ED. The dedicated TCN was an ED nurse with 6 years of working experience in the study hospital. Before initiating the protocol, she received 1 month of training in geriatric emergency medicine and home healthcare.

**Figure 1:**
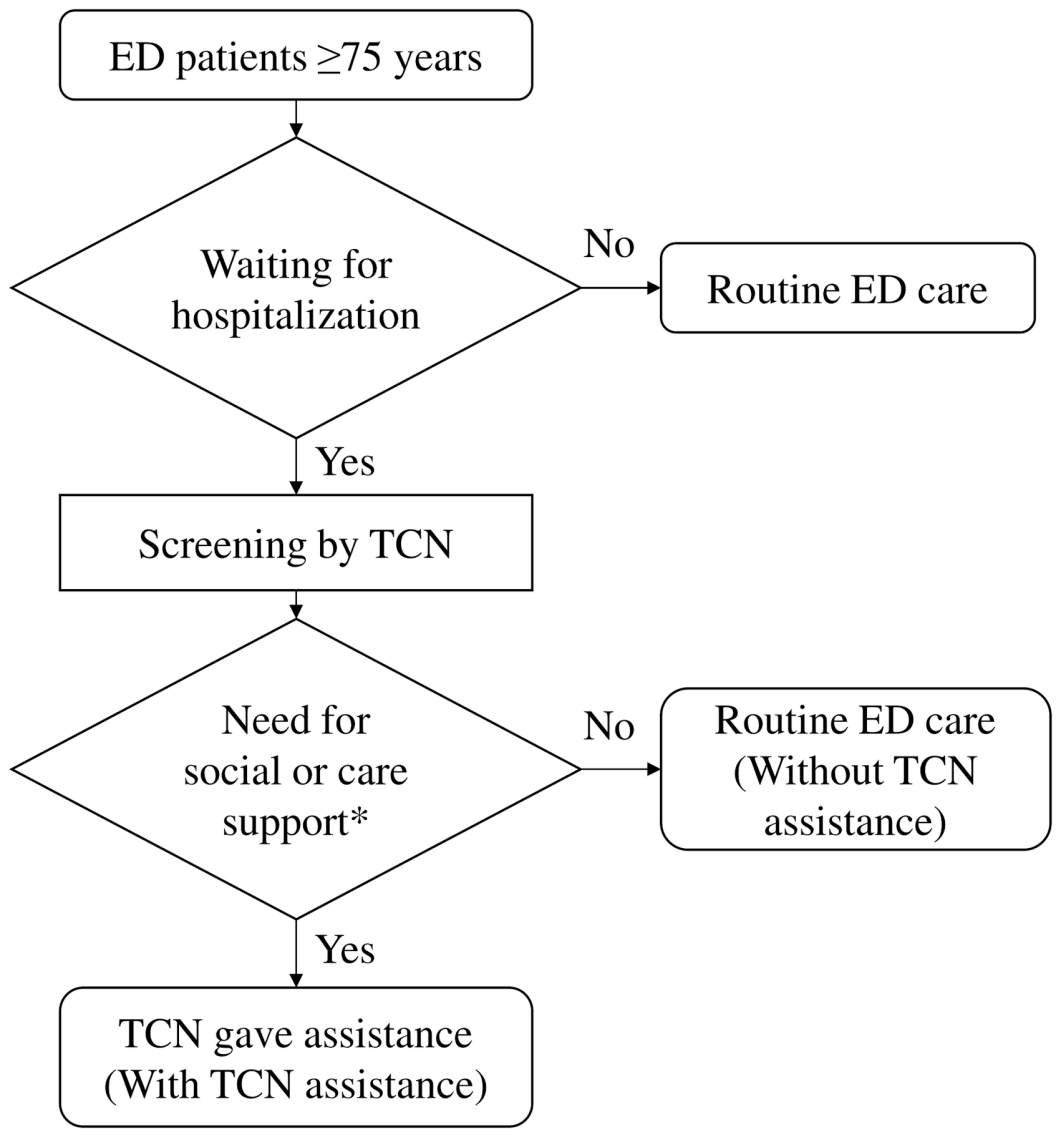
Flowchart of the protocol for ED transitional care. ED, emergency department; TCN, transitional care nurse.

Second, following a consensus among the interdisciplinary team, a flowchart outlining the protocol for ED TC was developed (Figure 1). A TCN screened patients who were aged 75 years or older and waiting for hospitalization in the ED to assess their need for social or care support. The TCN’s working hours were from 8:00 am to 5:00 pm on weekdays, Monday through Friday. Patients who met the screening criteria were randomly visited by the TCN, and those in need of social or care support were classified as the cohort with TCN assistance. Conversely, those who did not require such support were classified as the cohort without TCN assistance. The TCN evaluated patients for social or care support needs across five different aspects. The first aspect focused on providing home care instructions to foreign caregivers, including assessing their abilities and guiding them on nursing skills. The second aspect pertained to providing care instructions to family members of older adults residing in long-term facilities, which involved assessing their abilities and providing nursing skill guidance. The third aspect of the TCN’s assistance involved assessing the patient’s daily activities and long-term care needs. If necessary, the TCN referred the patient to a long-term care institution or nursing home. Additionally, the TCN provided information and assistance with applications for long-term care services, including transportation services, assistive device rental or purchase, home nursing, respite care services, day care centers, and other related services. The fourth aspect of the TCN’s role was to consult with a social worker to assist patients with financial, emotional, and social support issues. However, it should be noted that financial assistance was only considered successful when patients met the conditions for social welfare subsidies. The fifth aspect involved evaluating and arranging for home healthcare of the patient after hospital discharge if appropriate. Telephone follow-up was conducted with all patients after their hospital discharge.

Thirdly, a computer-assisted case management system was developed for ED TC, which integrated patients’ medical information, electronic assessment forms, and the aspects of social and care needs that were addressed by the TCN.

Fourth, all ED staff members were trained on the ED TC protocol, and the implementation of this protocol was officially announced on March 1, 2022.

### 2.3 Data collection: retrospective cohort study

**Figure 2:**
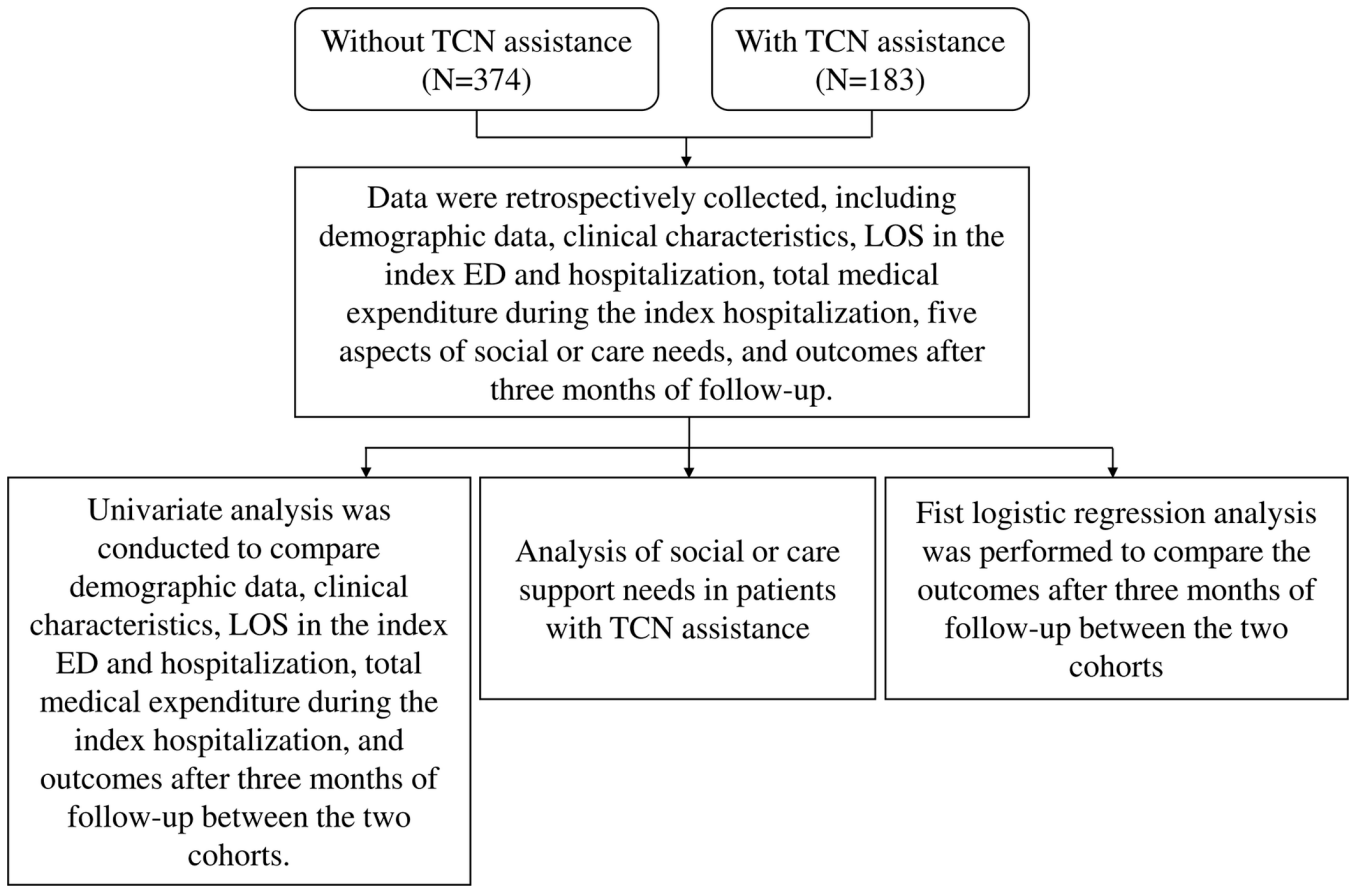
Flowchart of the study design. TCN, transitional care nurse; LOS, length of stay; ED, emergency department.

Retrospective data of patients who were screened by TCN from March to October 2022 were collected. Patients who received with the five aspects of social or care need support by TCN were classified as the cohort with TCN assistance, while those who did not were classified as the cohort without TCN assistance (Figure 2). Data was collected by an experienced ED TCN, who was blinded to patient outcomes.

### 2.4 Characteristics and outcome comparisons between two cohorts

To investigate the characteristics of the population requiring social or care support (referred to as the cohort with TCN), we conducted a comparison between two cohorts. The comparison focused on clinical characteristics, length of stay (LOS) in the index ED and hospitalization, and total medical expenditure during the index hospitalization. The ED triage was conducted using the Taiwan Triage and Acuity Scale (TTAS), a modification of the Canadian Emergency Department Triage and Acuity Scale (16), with the following categories: (1) Category 1: critical conditions requiring immediate life-saving interventions, (2) Category 2: emergent conditions requiring prompt medical attention to prevent serious outcomes, (3) Category 3: urgent conditions that are not life-threatening but require timely evaluation and treatment, (4) Category 4: less urgent conditions where delays in care would unlikely cause harm, and (5) Category 5: non-urgent conditions suitable for outpatient management. Additionally, an analysis was performed on five aspects of social or care needs in patients with TCN. Both cohorts were followed up for 3 months to compare outcomes, which included total medical expenditure within 3 months after hospital discharge, ED visit within 3 days after hospital discharge, re-hospitalization within 14 days after hospital discharge, and death within 1 month after hospital discharge.

### 2.5 Ethical statements

Following approval from the institutional review board of the study hospital, this retrospective study was conducted using anonymized patient data in accordance with the principles outlined in the Declaration of Helsinki. Informed consent was waived due to the study’s retrospective design, which posed no risk to patient welfare.

### 2.6 Statistics

In the univariate analysis, categorical variable analysis was conducted using the Chi-Squared Test or Fisher’s exact test, while continuous variable analysis utilized the Mann–Whitney U Test. Fist logistic regression analysis was performed to compare the outcomes between the two cohorts. Statistical analyses were carried out using Statistical Analysis System 9.4 (SAS Institute Inc., Cary, NC, United States). A significance level of 0.05 (two-tailed) was employed.

## 3 Results

We successfully implemented an ED TC model, screening a total of 557 older ED patients. Of these, 183 patients received TCN assistance, while 374 patients did not (Table 1). Compared to patients without TCN, those with TCN assistance were older (84.0 years vs. 81.0 years, *p* < 0.001), had a higher percentage of individuals aged ≥85 (47.0% vs. 28.3%, p < 0.001), a lower body mass index (22.5 vs. 23.5, *p* = 0.034), and a lower Glasgow Coma Scale (13.4 vs. 14.4, *p* < 0.001). Most patients in both cohorts were triaged as either category 2 or 3, but the TCN cohort had a higher proportion of patients triaged as category 1 (7.7% vs. 6.2%) and category 2 (48.6% vs. 31.3%), along with a lower proportion of patients triaged as category 3 (43.7% vs. 62.6%) compared to the cohort without TCN (*p* < 0.001). No patients in either cohort were triaged as category 4 or 5. Most patients presented to the ED for non-traumatic reasons, and there was no difference between the two cohorts in this regard. The TCN cohort had a higher proportion of underlying comorbidities and iatrogenesis compared to the cohort without TCN assistance. These included congestive heart failure (26.8% vs. 17.7%, *p* = 0.012), cerebrovascular disease (51.4% vs. 34.8%, *p* < 0.001), dementia (41.5% vs. 14.7%, *p* < 0.001), diabetes (33.9% vs. 23.3%, *p* = 0.008), nasogastric feeding (26.8% vs. 5.9%, *p* < 0.001), Foley indwelling (37.2% vs. 18.7%, *p* < 0.001), bedridden status (87.4% vs. 59.1%, *p* < 0.001), and receiving hospice and palliative care (37.7% vs. 17.7%, *p* < 0.001). In terms of ED diagnosis or symptoms/signs, the TCN cohort had a higher proportion of urinary tract infections (18.6% vs. 10.4%, *p* = 0.007) and pneumonia (16.9% vs. 9.4%, *p* = 0.009) compared to the cohort without TCN. Additionally, the TCN cohort had a longer LOS in the index ED visit (median: 24.8 h vs. 31.0 h, *p* < 0.001), longer LOS in the index hospitalization (median: 10 days vs. 7 days, *p* < 0.001), and higher total medical expenditure during index hospitalization (median: 76,532.0 New Taiwan Dollars [NTD] vs. 58,678.5 NTD, *p* = 0.001) compared to the cohort without TCN.

**Table 1:** Comparison of demographic data and clinical characteristics between older ED patients without and with TCN assistance.

Variables	Without TCN assistance (*n* = 374)	With TCN assistance (*n* = 183)	*p*-value*
Sex			0.509
Female	183 (48.9)	95 (51.9)	
Male	191 (51.1)	88 (48.1)	
Age (years)	81.0 (78.0–85.0)	84.0 (80.0–88.0)	<0.001
Age subgroup			<0.001
75–84 years	268 (71.7)	97 (53.0)	
≥85 years	106 (28.3)	86 (47.0)	
Body mass index	23.5 (20.7–25.8)	22.5 (20.3–25.0)	0.034
Vital signs			
Body temperature (°C)	36.6 (36.3–37.2)	37.1 (36.5–37.6)	<0.001
Heart rate (/min)	90.0 (76.0–102.0)	87.0 (74.0–105.0)	0.542
Respiratory rate (/min)	17.0 (16.0–19.0)	18.0 (16.0–20.0)	0.002
Systolic blood pressure (mmHg)	152.5 (125.0–179.0)	153.0 (126.0–177.0)	0.806
Glasgow coma scale	14.4 ± 2.0	13.4 ± 2.7	<0.001
ED triage†			<0.001
1	23 (6.2)	14 (7.7)	
2	117 (31.3)	89 (48.6)	
3	234 (62.6)	80 (43.7)	
4&5	0	0	
Laboratory data			
White blood cell count (10^3/uL)	8.4 (6.2–11.5)	9.1 (6.2–11.8)	0.396
Hemoglobin (g/dL)	11.8 (10.1–13.2)	11.0 (9.6–12.8)	0.023
Platelet (1,000/μL)	193.0 (153.0–245.0)	195.0 (158.0–243.0)	0.551
Serum creatinine (mg/dL)	1.1 (0.8–1.6)	1.1 (0.8–1.8)	0.877
Trauma or non-trauma			0.124
Non-trauma	339 (90.6)	158 (86.3)	
Trauma	35 (9.4)	25 (13.7)	
Underlying comorbidity			
Myocardial infarction	37 (9.9)	15 (8.2)	0.518
Congestive heart failure	66 (17.7)	49 (26.8)	0.012
Cerebrovascular disease	130 (34.8)	94 (51.4)	<0.001
Dementia	55 (14.7)	76 (41.5)	<0.001
Chronic pulmonary disease	123 (32.9)	72 (39.3)	0.134
Diabetes	87 (23.3)	62 (33.9)	0.008
Chronic kidney disease	97 (25.9)	62 (33.9)	0.051
Malignancy	127 (34.0)	56 (30.6)	0.428
Iatrogenesis (%)			
Nasogastric feeding	22 (5.9)	49 (26.8)	<0.001
Foley indwelling	70 (18.7)	68 (37.2)	<0.001
Tracheostomy	3 (0.8)	4 (2.2)	0.225
Bedridden	221 (59.1)	160 (87.4)	<0.001
Hospice and palliative care (%)	66 (17.7)	69 (37.7)	<0.001
ED diagnosis or symptom/sign			
Urinary tract infection	39 (10.4)	34 (18.6)	0.007
Fever	25 (6.7)	20 (10.9)	0.084
Delirium	2 (0.5)	2 (1.1)	0.601
Hyponatremia	11 (2.9)	8 (4.4)	0.382
Pneumonia	35 (9.4)	31 (16.9)	0.009
Weakness	15 (4.0)	10 (5.5)	0.436
Acute kidney injury	18 (4.8)	6 (3.3)	0.402
Limb cellulitis	5 (1.3)	5 (2.7)	0.309
Hyperglycemia	5 (1.3)	2 (1.1)	>0.999
Vertigo	13 (3.5)	0	0.007
LOS in the ED/h	24.8 (7.6–39.8)	31.0 (17.1–46.8)	<0.001
LOS in hospital/day	7.0 (4.0–11.0)	10.0 (6.0–16.0)	<0.001
Total medical expenditure during index hospitalization (New Taiwan Dollars)	58678.5 (34839.0–111206.0)	76532.0 (46840.0–144683.0)	0.001

Data were presented as median (Q1 − Q3) or n (%). *Categorical variables analysis by the Chi-Squared Test or Fisher’s exact test and continuous variables analysis by the Mann–Whitney U Test. †We used the Taiwan Triage and Acuity Scale for the ED triage, which was modified from the Canadian Emergency Department Triage and Acuity Scale. ED, emergency department; TCN, transitional care nurse; LOS, length of stay.

Among patients with TCN assistance (Table 2), the most common social or care need aspect was “providing home care instructions to a foreign caregiver (38.4%),” followed by “long-term care referral (30.0%),” “providing care instructions to a family member of an older adult living in a long-term care facility (26.3%),” “social worker referral (3.2%),” and “home healthcare referral (2.1%)”.

**Table 2:** Aspects for need of social or care support in the older ED patients who received TCN assistance.

Aspects	Frequency (*n* = 190)*	%
1. Providing home care instructions to a foreign caregiver	73	38.4
2. Providing care instructions to a family member of an older adult living in a long-term care facility	50	26.3
3. Long-term care referral	57	30.0
4. Social worker referral†	6	3.2
5. Home healthcare referral	4	2.1

ED, emergency department; TCN, transitional care nurse. *One patient might have multiple aspects. †Referring individuals to a social worker for assistance with a variety of issues such as financial, emotional, or social support.

In the univariate analysis, the cohort with TCN assistance had a higher total medical expenditure <3 months after hospital discharge (median: 126,427.0 NTD vs. 89,093.5 NTD, *p* = 0.004) compared to the cohort without TCN (Table 3). However, when utilizing Fist logistic regression analysis with adjustment for confounding factors (Table 4), there was no significant difference found in total medical expenditure <3 months after hospital discharge, ED visit ≤3 days after hospital discharge, re-hospitalization ≤14 days after hospital discharge, and death <1 month after hospital discharge between the two cohorts.

**Table 3:** Outcomes in older emergency department patients after hospital discharge: a univariate analysis comparing patients with and without TCN assistance.

Outcome	Without TCN assistance (*n* = 374)	With TCN assistance (*n* = 183)	*p*-value*
Total medical expenditure <3 months after hospital discharge (New Taiwan Dollars)	89093.5 (54134.0–215238.0)	126427.0 (68019.0–266733.0)	0.004
ED visit ≤3 days after hospital discharge	19 (5.1)	10 (5.5)	0.848
Re-hospitalization ≤14 days after hospital discharge	31 (8.3)	24 (13.1)	0.073
Death <1 month after hospital discharge	2 (0.5)	1 (0.6)	>0.999

Data were presented as median (Q1 − Q3) or *n* (%). *Categorical variables analysis by the Fisher’s exact test, continuous variables analysis by the Mann–Whitney U Test. TCN, transitional care nurse; ED, emergency department.

**Table 4:** Comparison of outcomes in older emergency department patients after hospital discharge: first logistic regression analysis comparing patients with and without TCN assistance.

Outcome (with TCN assistance vs. without TCN assistance)	Crude OR (95% CI)	*p*-value	Adjusted OR* (95% CI)	*p*-value
Total medical expenditure <3 months after hospital discharge (New Taiwan Dollars)				
<98,362†	Reference		Reference	
≥98,362	1.78 (1.24–2.54)	0.002	1.48 (0.96–2.28)	0.077
ED visit ≤3 days after hospital discharge				
No	Reference		Reference	
Yes	1.08 (0.49–2.37)	0.848	0.60 (0.23–1.56)	0.291
Re-hospitalization ≤14 days after hospital discharge				
No	Reference		Reference	
Yes	1.67 (0.95–2.94)	0.075	1.00 (0.51–1.97)	0.999
Death <1 month after hospital discharge				
No	Reference		Reference	
Yes	1.02 (0.09–11.35)	0.986	0.50 (0.04–6.57)	0.595

*Multiple models adjusted by sex, age, body mass index, triage, trauma or non-trauma, myocardial infarction, congestive heart failure, cerebrovascular disease, diabetes, dementia, cerebrovascular disease, coronary artery disease, chronic kidney disease, chronic obstructive pulmonary disease, iatrogenesis, bedridden. †Using median of the total medical expenditure <3 months as the cut-off point. TCN, transitional care nurse; ED, emergency department; OR, odds ratio; CI, confidence interval.

## 4 Discussion

We successfully implemented an ED TC model for older patients through collaboration among an interdisciplinary team and a dedicated TCN. Our findings revealed that patients who received TCN assistance exhibited greater frailty, as indicated by older age, higher acuity, a higher proportion of underlying comorbidities and receiving hospice and palliative care, longer LOS in the index ED visit and hospitalization, and higher total medical expenditure during the index hospitalization (Table 1). The most common aspect requiring social, or care support was “providing home care instructions to a foreign caregiver,” followed by “long-term care referral” and “providing care instructions to a family member of an older adult living in a long-term care facility (Table 2)”. However, despite these factors, the cohort with TCN did not demonstrate inferior outcomes compared to the cohort without TCN (Table 4).

The success of this model relies on a dedicated TCN and effective collaboration among interdisciplinary team members. Having a dedicated healthcare professional for TC in the ED is considered a beneficial geriatric care model (17). A systematic review has demonstrated that dedicated healthcare professionals have two primary functions: conducting individual needs assessments of ED patients and coordinating discharge planning and services (17). Implementing a structured individual needs assessment has been associated with a significant decrease in hospital admissions, hospital readmissions, and ED revisits (17). Additionally, developing individualized discharge plans from the ED has shown a significant decrease in ED revisits and hospital readmissions (17). Our model essentially embodies a structured individual needs assessment. Given the complex nature of issues faced by older patients, geriatric integrated care necessitates interdisciplinary or multidisciplinary teamwork (17). The specific components of the structured individual needs assessment and the composition of the interdisciplinary team depend on the objectives of the hospital, the needs of the patient/caregiver, and the available local resources (4, 17).

This study examined the characteristics of patients who received TCN assistance, aiming to identify the target population in need of social and care support in the future (Tables 1, 2). The screening and inclusion criteria for TCN assistance depend on the hospital’s objectives, available local resources, and the population likely to experience better outcomes (4, 5, 18). The first implementation of the TCN model was carried out through the program called “GEDI WISE” in the United States (5). In the GEDI WISE program, TCN was used to screen ED patients aged 65 and above and assess their cognitive function, delirium, functional status, falls risk, care transitions, and caregiver strain (5). The choice of assessments used in the GEDI WISE program was based on preexisting hospital programs or staff preferences in the anticipated three hospitals (5). In a randomized controlled trial, ED patients aged 65 and above who revisited the ED within 30 days were included to evaluate the effects of an ED-to-home TC program on outcomes (18). This study classified the patients into six classes and observed differences in outcomes among these classes (18).

Our model assessed five aspects of the services provided by the TCN and revealed that the most common social or care support was the “providing home care instructions to foreign caregivers” (Table 2). In 2022, approximately 30% of foreign workers in Taiwan were employed as caregivers, with over 90% providing round-the-clock live-in care services (19), highlighting the high demand for foreign caregivers in Taiwan. However, foreign caregivers often face language barriers and lack medical care education, necessitating the providing home care instructions by the TCN, as demonstrated in this study. The second most prevalent social or care need identified in this study was long-term care referral. Since 2017, the Taiwanese government has expanded the scope of long-term care services and allocated increased funds to promote Long-term Care 2.0 (20). Nevertheless, most people lack knowledge about long-term care services and the application process, prompting the TCN program to assist patients and their families in understanding and applying for such services. In 2021, approximately 52,000 individuals aged 65 or above resided in long-term care facilities in Taiwan, representing around 1.3% of the older population (21). This option provides an alternative to informal care by family members or foreign caregivers. However, even when older adults live in long-term care facilities, their family members may still require coordination with the facility staff, thus highlighting their need for social or care support. The proportions of social worker referrals and home healthcare referrals were notably low. The low proportion of social worker referrals primarily resulted from most patients failing to meet the government’s social welfare subsidy requirements, leading to unsuccessful referrals. The low rate of home healthcare referrals may be attributed to the shortage of home healthcare professionals in the study hospital. Since 2020, the hospital has initiated ED referrals for home healthcare, which have shown lower median total medical expenditure within 3 months compared to home healthcare initiated after hospitalization (14). In the future, our aim is to expand the service by recruiting more healthcare professionals to meet the needs of this population.

The univariate analysis revealed higher medical expenditures within 3 months of hospital discharge among patients with TCN assistance compared to those without, likely due to their inferior underlying condition (Table 3). However, after adjusting for confounding factors, the difference was not statistically significant (Table 4). These findings demonstrate a promising result for TCN assistance in our model.

One major strength of our study is the successful implementation of a novel TCN model in Taiwan, which can serve as an important reference for the future promotion of TCN in the ED. However, the study has several limitations. Firstly, the retrospective design and small sample size limit the generalizability of the findings. To address this limitation, a prospective randomized study with a larger sample size should be conducted in the future to provide further clarification on the topic. Another limitation is that the applicability of this model may be limited to the specific context of our hospital and may not fully generalize to other hospitals or nations. This is due to variations in hospital objectives and available local resources. Therefore, modifications would be necessary if implementing this model in other healthcare settings.

## 5 Conclusion

Our study successfully implemented a TC model for older patients in the ED. We observed that the population requiring TCN assistance tended to be older and had a more compromised condition compared to those who did not require TCN assistance. The most common needs for TCN assistance were providing home care instructions to foreign caregivers, facilitating long-term care referrals, and offering care instructions to family members of older adults residing in long-term care facilities. Upon adjusting for confounding factors, we found that patients receiving TCN assistance did not exhibit inferior outcomes compared to those without TCN, indicating the potential benefits of TCN assistance. This study has effectively identified the target population in need of TCN assistance, and our novel TC model can serve as a valuable reference for future implementation of TC in ED settings. However, it is important to note that further research using a prospective randomized design and larger sample sizes is necessary to explore the full impact of the TCN program. This future research should aim to assess the potential of TCN in reducing healthcare costs and improving outcomes for older ED patients.

## Data Availability

The datasets presented in this article are not readily available because the data that support the findings of this study are not publicly available due to privacy and institutional policies. Requests to access the datasets should be directed to jasonhuang0803@gmail.com.
